# Correction to “Intravaginal electrical stimulation: A randomized, double‐blind study on the treatment of mixed urinary incontinence”

**DOI:** 10.1111/aogs.14782

**Published:** 2024-02-05

Amaro, J.L., Gameiro, M.O., Kawano, P.R. and Padovani, C.R. (2006). Intravaginal electrical stimulation: a randomized, double‐blind study on the treatment of mixed urinary incontinence. Acta Obstetricia et Gynecologica Scandinavica, 85: 619–622. https://doi.org/10.1080/00016340500495058.

In this article the authors had not clearly stated that the conclusions are based on data from the same study population that was used for their previous research, published in 2005 in the *International Urogynecology Journal*,^1^ although the focus of the investigation was different. The article published in *Acta Obstetricia et Gynecologica Scandinavica* reports on the effect of intravaginal electrical stimulation therapy on mixed urinary incontinence, whereas the paper referenced above^1^ presents data on the effect of intravaginal electrical stimulation on pelvic floor muscle strength.

The authors and Chief Editor, Ganesh Acharya, agreed to this correction statement.
